# A Handbook of Antiseptics

**Published:** 1918-02

**Authors:** 


**A Handbook of Antiseptics.** Henry Drysdale Dakin, D.Sc., F. I. C., F. R. S., and Edward Kellogg Dunham, M. D., Emeritus Professor of Pathology, University and Bellevue Hospital Medical College, Major M. R. C. Cloth, 4¼x5¾ inches, ix + 129 pages, price $1.25. New York, The McMillan Company, 1917.

This little handbook, easily carried in the coat pocket, brings to a focus the experiences of the Great War in the use of chemical antiseptics. It is limited to describing the preparation and use of those old and new antiseptics that have found favor with military surgeons during the past three years. While thoroughly scientific and technical it is clear and practical, equally useful in the laboratory, at the bedside, in sterilizing drinking water and disinfecting hospital ships. After a general treatment of the laws and processes of disinfection, and the choice of antiseptics, the various chemical antiseptics are taken up in detail, about one-third of the space being devoted to the chlorine antiseptics, including Dakin's solution and the preparations related closely to it. A very useful chapter presents the methods of testing antiseptics.. The last chapter gives directions for disinfection of hospital ships with hypochlorite prepared by electrolysis of sea water. A dozen blank pages are added for notes. This is just the book for a busy man.—F. W. B.

